# Recent Advances in Nanostructured Conversion-Type Cathodes: Fluorides and Sulfides

**DOI:** 10.3390/nano15060420

**Published:** 2025-03-08

**Authors:** Mobinul Islam, Md. Shahriar Ahmed, Sua Yun, Basit Ali, Hae-Yong Kim, Kyung-Wan Nam

**Affiliations:** 1 Department of Energy & Materials Engineering, Dongguk University, Seoul 04620, Republic of Korea; shahriar.emcl@dgu.ac.kr; 2 Department of Advanced Battery Convergence Engineering, Dongguk University, Seoul 04620, Republic of Korea; 3 Department of Chemistry and Materials Science, Aalto University, FI-00076 Aalto, Finland; basit.ali@aalto.fi

**Keywords:** nanostructure, conversion cathode, conversion anode, lithium-ion batteries, nanomaterials, nanoparticle

## Abstract

This review paper explores the emerging field of conversion cathode materials, which hold significant promises for advancing the performance of lithium-ion (LIBs) and lithium–sulfur batteries (LSBs). Traditional cathode materials of LIBs, such as lithium cobalt oxide, have reached their limits in terms of energy density and capacity, driving the search for alternatives that can meet the increasing demands of modern technology, including electric vehicles and renewable energy systems. Conversion cathodes operate through a mechanism involving complete redox reactions, transforming into different phases, which enables the storage of more lithium ions and results in higher theoretical capacities compared to conventional intercalation materials. This study examines various conversion materials, including metal oxides, sulfides, and fluorides, highlighting their potential to significantly enhance energy density. Despite their advantages, conversion cathodes face numerous challenges, such as poor conductivity, significant volume changes during cycling, and issues with reversibility and stability. This review discusses current nanoengineering strategies employed to address these challenges, including nano structuring, composite formulation, and electrolyte optimization. By assessing recent research and developments in conversion cathode technology, this paper aims to provide a comprehensive overview of their potential to revolutionize lithium-ion batteries and contribute to the future of energy storage solutions.

## 1. Introduction

Batteries can be grouped into different types: either primary or secondary. After their chemical energy is depleted, primary batteries cannot be recharged. Examples include zinc–carbon (Zn–C) cells and alkaline zinc–manganese dioxide (Zn–MnO_2_) cells. In contrast, secondary batteries are rechargeable and designed for repeated use. Common instances include lead–acid batteries, nickel–metal hydride (NiMH) batteries, nickel–cadmium (Ni-Cd) batteries, lithium-ion batteries (LIBs), sodium-ion batteries, lithium–sulfur batteries (LSBs), magnesium-ion batteries (MIBs), and aluminium-ion batteries [1,2,3]. Among these, LIBs and LSBs are the favored option due to various benefits, such as superior energy density, longevity, and cost efficiency. Their high energy density enables them to store substantial energy in a compact and lightweight structure, making them well-suited for portable electronics like smartphones and laptops. LIBs have an especially long lifespan, capable of enduring hundreds of charges–discharges cycles with minimal capacity degradation, which enhances their long-term cost-efficiency [3]. Other notable features include their low self-discharge rate, enabling them to retain a charge for extended periods when not in use, and their immunity to the memory effect, which eliminates the need for complete discharge before recharging. LIBs also support rapid recharging due to their high charge acceptance rate. These attributes, combined with their portability and lightweight design, make LIBs invaluable in uses such as sustainable energy storage systems, electric vehicles, and consumer electronics [4]. In addition, a compact, high-energy density, lightweight, and seamlessly integrable energy storage device is essential for self-sufficient internet of things (IoT) devices at remote edges, enabling independent operation in miniaturized applications like smart cards, medical devices, military equipment, and electronics [2]. The versatile design of LIBs has made them the dominant choice in that kind of consumer and industrial application. On the other hand, LSBs are promising post-lithium-ion battery technology because their energy density is maximum for various applications. For this reason, multidisciplinary research is ongoing to develop fundamental understanding, modeling, and application-based control of LSBs over the past decade.

LIBs comprise four major parts: cathode, anode, electrolyte, and separator. Each component joins in a specific role in ensuring optimal performance, safety, and longevity. LSBs are also constructed by using similar prototypes. Among these components, the cathode extends as a major contributor to the overall energy storage, capacity, and lifespan of the battery [3]. Its composition directly affects the energy that the battery can reserve and carry, as well as its stability during repeated cycling [5]. During the charging process, oxidation occurs at the cathode, causing electrons to flow from the cathode to the anode via an external circuit. Simultaneously, lithium ions (Li⁺) are released from the cathode and travel through the electrolyte to the anode, where they are intercalated into the anode host. During discharge, the procedure is inverted, with lithium ions and electrons moving back to the cathode. The separator ensures that ions transfer without short-circuiting by blocking direct electron flow. This reversible insertion (intercalation) and extraction (de-intercalation) of lithium ions moving in and out of the host materials are the basis for LIB operation. This mechanism has earned LIBs the nicknames “rocking chair batteries” or “swing batteries” [6].

The advancement of high-performance cathode materials continues to pose a substantial challenge due to conflicting requirements such as cost, toxicity, stability, etc. Numerous materials have been researched as cathodes and anodes for LIBs, acting as host structures for the insertion and extraction of Li⁺ ions. The effectiveness of these materials is contingent upon their architectural integrity and the recoverability of Li⁺ insertion/extraction during cycles. The suitability of a material for a cathode or anode depends on its redox potential for lithium insertion/extraction. Essential criteria for LIB electrode materials encompass reversible Li⁺ intercalation, high cell voltage, good electronic conductivity, structural stability during cycling, high Li⁺ diffusivity for power density, and cost-effectiveness [7]. Intercalation compounds, such as metal oxides, sulfides, and selenides, are particularly effective for electrode applications [8]. Transition metal compounds are widely used as cathodes due to their variable oxidation states, which balance charges during lithium exchange. The performance of the cathode is greatly impacted by the transition metals integrated into its structure [9]. A variety of elements, such as cobalt (Co), iron (Fe), nickel (Ni), manganese (Mn), and aluminum (Al), have been used to enhance electrochemical properties and stabilize cathode structure. In the creation of Li-ion batteries, these metals determine the cathode’s performance traits, such as its capacity, voltage output, and thermal stability. Current state-of-the-art cathode materials, such as layered transition metal oxides, olivine structured polyanionic compound, and spinel phase metal oxides, have unique advantages and limitations. For example, layered oxides such as LiMO_2_ offer high energy density but are limited by voltage decay and structural instability during cycling [9,10]. Olivine materials, such as LiFePO_4_, exhibit excellent thermal stability but are hindered by low ionic and electronic conductivity [11,12]. Spinel structures provide high power density and stability but are prone to metal dissolution and rapid capacity fading [13]. In addition to intercalation compounds, conversion-type cathode materials based on multi-step conversion reaction mechanisms have been extensively documented for LIBs. Conversion-type cathode materials offer several advantages over commonly used intercalation-type cathodes. Transition metals like cobalt (Co), which is expensive and scarce, are typically used in intercalation cathodes. On the other hand, conversion-type cathodes can utilize elements like oxygen (O), sulfur (S), iron (Fe), copper (Cu), and others that are more abundant and less costly. These elements are also safer for the environment and human health. While pure halogens are hazardous, they become stable and environmentally friendly when incorporated into salt as part of conversion cathodes. Furthermore, conversion-type cathodes have a higher storage capacity, with typically 2–3 lithium ions being stored per individual chalcogen atom or transition metal, compared to 1.5–2 Co atoms required for intercalation cathodes [14,15].

Nanotechnology stands as a revolutionary science, with the capability to transform various industries by creating nanostructured materials with customized properties. With the advancement of nanotechnology, we have seen a paradigm shift in battery material design, resulting in new opportunities to achieve better performance from LIB cathodes [16,17]. Particularly, nanoscale anodes and cathodes provide short diffusion distances for rapid charging, increased electroactive sites for higher capacity, and longer lifespans due to the ability to endure volume changes during cycling [18]. In the past decade, nanotechnology has been a highly active research area, with numerous excellent reviews summarizing the various nanostructured cathode and anode materials for LIBs [19,20]. These studies shed light on intercalation-type electrode materials. In addition, nanoscale anode materials were extensively reviewed in several literature studies [21,22,23,24,25]. In a detailed review of conversion-type anode materials, Y. Lu et al. [26] emphasize the relationship between electrode performance and nanostructured morphology. However, there is a scarcity of articles providing a broad range of the detailed nanotechnology strategies used for conversion cathodes in LIBs. L. F. Olbrich et al. [27] discussed conversion-type transition metal fluoride cathodes in a review. They emphasized the importance of electrolyte optimization for improving cathode performance and stability. Additionally, they provided a realistic assessment of the rate capability and highlighted the challenges and potential solutions for enhancing the charge/discharge rates of conversion metal fluoride cathodes. Another review study aimed to compile various aspects, including the challenges in metal fluoride conversion cathodes and modification strategies like doping with other metal elements, surface modification, morphological regulation, and composite formation [28]. Instead, this review concentrated on linking nanoscale morphology with the performance of various metal fluorides and metal sulfide conversion cathodes. The purpose of this review is to give a critical overview of how high-performance conversion cathodes are being developed, including using nanostructures of reduced dimensionality, hierarchical porous nanostructured materials, exclusive hollow forms, and hybridizing with carbonaceous materials. This paper highlights the connection between the nanostructures of the conversion cathode produced and the enhanced electrochemical performance of the electrode.

## 2. Brief Overview of Conversion-Type Cathode Materials

Conversion cathode materials represent a distinct category of substances employed in rechargeable batteries, wherein they undergo a chemical conversion-type reaction during the charge and discharge processes. Distinguishing them from intercalation cathode materials that involve the reversible insertion and extraction of ions, conversion cathode materials undergo a more intricate transformation, resulting in a change in their material composition. A notable example of a conversion cathode material is iron fluoride (FeF_3_), which can undergo a reversible conversion between iron ions (Fe^2+^) and iron fluoride as the battery operates [29]. In addition to iron fluoride, common conversion cathode materials encompass various metal sulfides, metal fluorides, and metal phosphides [30,31]. These materials often exhibit high theoretical specific capacities, making them particularly appealing for applications demanding a high energy density.

The advancement of conversion-type cathode materials is significant for the advancement of upcoming rechargeable lithium (Li) and lithium-ion (Li-ion) power sources. It is imperative to make continuous and rapid progress in enhancing the performance of these cathodes in various aspects. This is essential to ensure their viability and effectiveness in future applications. The intercalation-type cathodes in commercial lithium-ion (Li-ion) batteries, which typically use nickel and cobalt, have several drawbacks. These include moderate energy, elevated toxicity, and high expense. Achieving a significant development of sustainability of energy capacity of these batteries is difficult due to the capacities of intercalation compounds approaching their theoretical limits [32,33]. Additionally, increasing the maximum voltage of these batteries raises serious safety concerns [34,35].

Conversion electrodes undergo breaking and form chemical bonds during the lithiation and delithiation processes. This procedure involves a solid-state redox reaction, with the fully reversible electrochemical reaction for conversion electrodes outlined as

Type A: MX_z_ + yLi↔M + zLi(y/z)X (1)

Type B: yLi + X_z_ ↔ Li_y_X (2)

**Figure 1:**
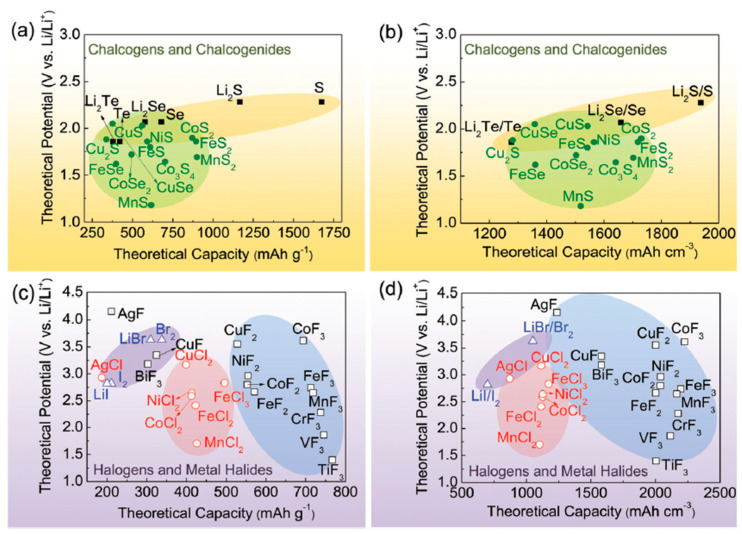
Selected conversion cathode materials with theoretical gravimetric and volumetric capacities, as well as theoretical potential: (**a**,**b**) chalcogens and chalcogenides; (**c**,**d**) halogens and metal halides, reproduced with permission from ref. [15]. Copyright 2017, Royal Society of Chemistry.

Unlike intercalation cathodes, conversion materials undergo a process of breaking and forming new chemical bonds when lithium (Li) is inserted or extracted. There are two distinct types of conversion reactions that can be identified in Li chemistries [15]. Type A, known as “true conversion”, is represented by the equation M^0^X_z_ + yLi_2_M + zLi(y/z)X. In this reaction, M^0^ represents the cation, M represents the reduced cation material, X_z_ represents the starting anion, and X represents the resulting product. Type A cathodes typically involve transition metal ions such as Fe^3+^, Fe^2+^, Ni^2+^, Cu^2+^, Co^2+^, Mn ^3+^, etc., while X_z_ typically represents halogen ions (F^−^, Cl^−^, Br^−^, and I^−^) or chalcogenide ions (S^2−^, Se^2−^, etc.). Type B, referred to as a “chemical transformation”, is described by the equation yLi + X_z_ → 2Li_y_X (2). In this reaction, yLi represents lithium atoms, X_z_ represents the starting anion, and X represents the resulting product. Type B reactions involve a direct interaction between lithium and the anion, leading to the creation of the desired materials. These two types of conversion reactions play a vital role in the behavior of conversion-type cathodes during charging. Metal halides are converted into metallic form by the addition of multiple lithium ions per metal atom, resulting in significantly higher theoretical capacities. This conversion reaction involves changes in structure between lithiated and unlithiated states, such as S and Li_2_S, Se and Li_2_Se, Te and Li_2_Te, Br_2_ and LiBr, I_2_ and LiI, etc., and these transformations also demonstrate remarkable theoretical capacities, which are shown in Figure 1.

**Scheme 1:**
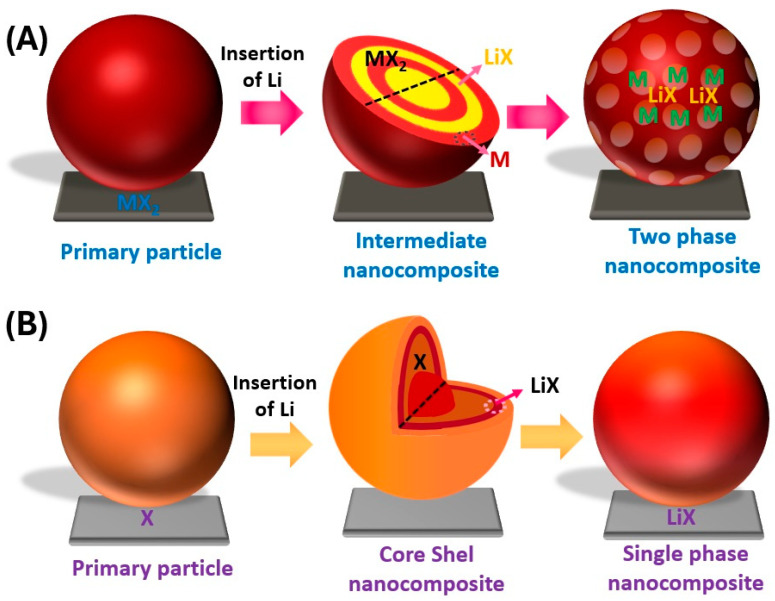
Type A and type B conversion reactions: (**A**) true conversion with the emergence of two distinct new phases; M = metals and X = halide anions. (**B**) Chemical transformation with the formation of a single new phase; X = S, Se, Te, Br, and I.

In summary, the reduction of metal halides to metallic states involves multiple lithium ions per metal atom, leading to increased capacities, and the structures undergo transitions between lithiated and unlithiated states. The structure and spatial arrangement of the newly formed phases can be influenced by various factors, including the diffusion coefficients of cations and anions, electronic and ionic conductivity of the new phases, and interfacial energies. For instance, when FeF_2_ undergoes lithiation, metallic Fe nanoparticles may form and remain in their original positions within the phase. On the other hand, the formed LiF tends to occupy the empty spaces surrounding the metallic Fe nanoparticles. This behavior can be attributed to the lower mobility of Fe^2+^ compared to Li^+^ and F^−^ [36], which causes Fe^2+^ to have a more limited movement within the structure. During lithiation, type A cathode particles undergo a conversion process, converting into nanocomposite particles consisting of interconnected metal M nanoparticles distributed within a Li(y/z)X matrix (Scheme 1A). In contrast, the lithiation of S, Se, Te, Br, and I involves a type B conversion reaction, also known as a “chemical transformation reaction”, which converts one single phase into a different single phase (Scheme 1B).

### 2.1. Limitations of Conversion Cathode

Conversion cathode materials encounter significant issues, including low conductivity, voltage hysteresis, substantial volume changes, and unfavorable interactions with electrolytes, all contributing to poor performance and efficiency [30]. All metal fluorides, metal chlorides, sulfur, lithium sulfide, iodine, bromine, lithium iodide, and lithium bromide are natural insulators with low electronic conductivity, apart from selenium and tellurium, which are semiconductors with relatively high conductivities [36,37,38,39,40,41,42]. These substances typically form ionic compounds, in which the metal and anion form strong bonds that do not allow electrons to move easily. This is why they are poor conductors of electricity.

In addition, the dissolution of materials causes capacity fading, soluble species shuttle, unrestrained re-formation, ionic pathway obstruction, a rise in cell resistance, and precipitation on the anode, potentially affecting stability and SEI properties, resulting in irreversible Li depletion and increased resistance [43]. In the case of CuF_2_, copper (Cu) undergoes electrochemical conversion to Cu^+^, which leads to the dissolution of the material into the electrolyte [44]. Moreover, just like many ionic compounds, metal fluorides are generally soluble in polar solvents. However, metal chlorides are more prone to dissolution in various solvents, including those commonly used in lithium battery electrolytes [45]. Additionally, certain electrolyte decomposition reactions catalyzed by conversion materials can cause gas formation and undesirable resistance increase [46,47]. Even in the absence of such undesired side reactions, metal nanoparticles tend to agglomerate during cyclic testing, exacerbating voltage hysteresis [48]. Lastly, when it comes to volume expansion limitations, the commonly employed metal fluorides and chlorides have shown volume expansions ranging from 2% to 25%. Volume changes may cause structural damage to the electrodes. Cathodes experiencing significant volume changes during discharge–charge cycles will continuously expose a bare surface, thereby encouraging undesirable side reactions [15,49,50]. Furthermore, the loss of electrical contact and the occurrence of cracking are additional factors that can diminish the electrical conductivity and ionic mobility of cathode materials. As a result, conversion cathode materials encounter significant challenges that impede their commercialization for lithium-ion batteries.

On the other side, lithium–sulfur (Li-S) batteries face several challenges that hinder their widespread adoption, including the low electrical conductivity of sulfur, which affects performance, and the polysulfide shuttle effect, where soluble polysulfides migrate between the cathode and anode, leading to capacity loss. Additionally, sulfur undergoes significant volume changes during cycling, causing structural degradation [51].

### 2.2. Strategies to Improve the Limitations of Conversion Cathode

Two primary approaches enhance the electrochemical performance of materials with less electronic and ionic conductivities, which are enhanced by reducing particle sizes to the nanoscale and uniformly blending them with a highly conductive matrix.

This combination provides efficient electrochemical performance. A successful example is a carbon-coated nanosized LiFePO_4_ cathode. The nanoscale size of the particles allows for the faster diffusion of lithium ions, while the carbon coating helps to increase conductivity and stabilize the cathode. The Li^+^ ion diffusion time t in solids is directly proportional to the square of the diffusion length, expressed as t = L^2^/2D, where D represents the Li^+^ ion diffusion coefficient [52]. The decrease in particle size shortens the diffusion time, resulting in a fast Li^+^ concentration equilibrium and low polarization. Consequently, it improves power performance, energy efficiency, and voltage hysteresis in conversion-type cathode materials. Charge transfer resistance might also be reduced due to their high external surface area [53]. The reversibility of lithium conversion reactions in metal halides is typically due to the formation of a connected nanoscale blend of metal and lithium halide clusters. Employing metal halide nanoparticles accelerates lithiation and reduces cluster dimensions [36]. Using CuF_2_ as an example, micro-sized CuF_2_ has a specific capacity of 100 mA h g^−1^ as opposed to 250 mA h g^−1^ for nanosized CuF_2_ [54].

Nanostructured materials with a high surface area have a high contact area with the electrolyte, surrounded by high Li^+^ flux. They also have more tolerance to stress and strain from local volume changes [55]. However, nano-scaling of conversion cathode material is not enough to compensate for volume expansion. The high surface area of nanosized active particles in electrolytes may increase undesirable side reactions, leading to self-discharge or faster cell degradation [56]. Another solution involves dispersing or wrapping active materials in conductive matrix materials to form composites with improved conductivity, such as FeF_3_/graphene [57], FeF_3_/CNT [58], and PEDOT-coated FeOF nanorods [59]. Hybrid nanocomposites, combining conversion cathode materials with functional media such as a conductive carbon matrix, are being developed to improve electrical conductivity, mechanical stability, and other performance metrics [38,40]. Various conductive carbons, including graphene, CNTs, carbon cloth, carbon fibers, and carbon blacks, have shown promising improvements in electrochemical performances for lithium-ion batteries. In addition to conductive carbons, conducting polymers, metal–organic frameworks, and various metal oxides (such as Fe_2_O_3_, Ti_4_O_7_, etc.) have also been employed to enhance the conductivities of S-based and metal fluoride-based cathodes and mitigate their dissolution during cycling [59,60,61,62].

**Figure 2:**
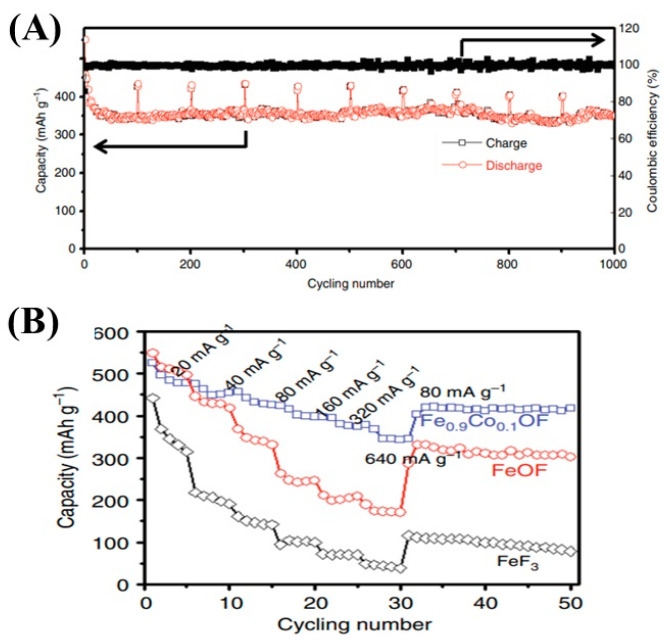
The electrochemistry of the Fe_0.9_Co_0.1_OF nanorod cathode. (**A**) The cycling performance at a current of 500 mA g^−1^. (**B**) Rate of the Fe_0.9_Co_0.1_OF cathode was compared to that of FeOF and FeF_3_ cathode materials, adapted from ref. [66], Nature Publication.

Conversion reaction-type materials suffer from sluggish phase transformation kinetics. The incorporation of both cation and anion dopants leads to a thermodynamic reduction in the potential for conversion reactions. For example, in iron fluoride, this shift causes a transition from the less reversible intercalation–conversion reaction observed to a significantly more reversible intercalation–extrusion reaction in iron fluoride that has undergone co-doping [63]. Jian Su et al. It was demonstrated that the electrochemical properties of the iron fluoride cathode, which are constrained by intrinsic slow kinetics and low electronic conductivity, could be greatly enhanced through non-equivalent cobalt doping [64]. Ghulam Ali et al. synthesized cobalt-doped iron fluoride (Fe_0.9_Co_0.1_F_3_∙0.5H_2_O) with a high discharge capacity of 227 mA h g^−1^ at 0.1 C in the potential range of 1.8–4.5 V versus Li/Li^+^ [65]. In another investigation conducted by Fan et al., they successfully developed a reversible iron fluoride (Fe_0.9_Co_0.1_OF) cathode with a high energy density by incorporating cobalt and oxygen into the iron fluoride structure through coordinated doping [66]. The resulting Fe_0.9_Co_0.1_OF cathode demonstrated an impressive energy density of 1000 W h kg^−1^ and maintained a long cycle life of 1000 cycles while retaining a capacity of 350 mA h g^−1^ throughout (Figure 2A). The rate capability of Fe_0.9_Co_0.1_OF, FeOF, and FeF_3_ cathode materials is shown in Figure 2B. It reveals that Fe_0.9_Co_0.1_OF exhibits a significantly higher rate capability compared to the other materials. The simultaneous substitution of cations and anions in the engineered conversion cathode material thermodynamically lowered the conversion reaction potential, transitioning the process from a less-reversible intercalation–conversion mechanism to a highly reversible intercalation–extrusion reaction.

Another critical aspect involves the adjustment of the electrolyte composition to minimize undesired interactions between the electrolyte and the active materials during various charging and discharging phases. A study conducted by Q. Huang et al. demonstrated an innovative solution to enhance the subpar performance of metal fluoride conversion cathodes at higher temperatures [67]. This solution involved substituting the conventional organic liquid electrolyte with a solid polymer electrolyte (SPE), resulting in improved cathode performance. Furthermore, their innovative strides led to the creation of a more lightweight, ecologically friendly, and cost-efficient battery. This was achieved by crafting a versatile cathode, where FeF_2_ nanoparticles and solid polymer electrolyte (SPE) were embedded within the interstices of a carbon nanotube (CNT) framework. The utilization of SPE played a pivotal role in curtailing electrolyte degradation, while the enhanced mechanical attributes of SPE bolstered the structural robustness of conversion cathodes. Notably, the development of a thin, pliable, and conformal solid electrolyte interface (CEI) on the surface of FeF_2_ particles in contact with SPE resulted in a remarkable escalation in rate performance. This was accompanied by a promising capacity exceeding 450 mA h g^−1^ and cyclic stability spanning over 300 cycles at 50 °C. Importantly, this composite material exhibited reduced side reactions and a diminished voltage hysteresis compared to its liquid electrolyte counterpart. These factors together facilitate the potential commercialization of metal fluoride batteries. However, a significant constraint of these cathode materials lies in their absence of a lithium composition. Addressing this limitation, recent advancements have emerged through the amalgamation of the transitional metal fluorides with lithium fluorides, leading to cathode materials that offer elevated capacity. Kim and colleagues successfully created LiF + FeF_2_ composites through effective blending, yielding a capacity of 190 mA h g^−1^ at a rate of 50 mA g^−1^ [68]. Intriguingly, X. Fan and his research group documented the achievement of well-integrated Fe + LiF + C and FeM + LiF + C (where M = Co, Ni) nanocomposites, showcasing impressive capacities surpassing 400 and 300 mA h g^−1^, respectively [69,70].

Over the past decade, the systematic implementation of nanotechnology across various components of lithium–sulfur (Li-S) cells has led to significant advancements in Li-S battery performance. Specifically, nanomaterials have been utilized to tackle the three primary challenges previously mentioned: the low electrical conductivity of sulfur and its sulfides, the shuttling effect caused by polysulfide dissolution, and issues related to volumetric expansion. By offering shorter pathways for ions and electrons and polysulfide confinement, nanomaterials effectively improve the performance of the cathode, addressing major key limitations in these batteries [71,72].

## 3. Fluoride Compounds

**Scheme 2:**
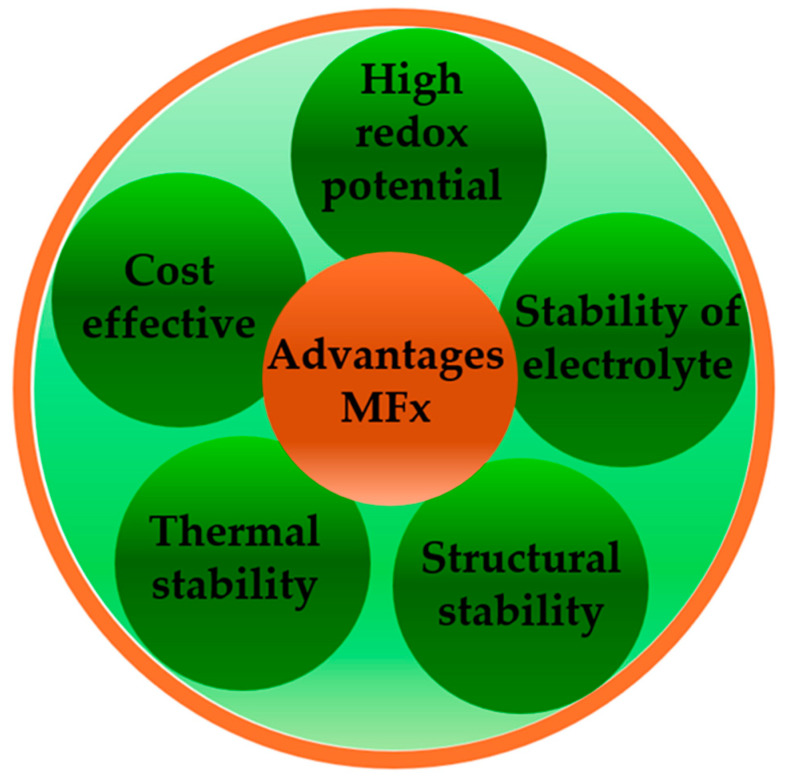
Advantages of MFx in high-performance LIBs.

Conversion cathode materials based on fluorine compounds have received significant attention in battery research due to their potential for high energy density and the advancement of rechargeable batteries (Scheme 2). However, these materials face several challenges that necessitate solutions. One major challenge arises from the high reactivity of fluorine compounds with the electrolyte and other battery components. This reactivity leads to the degradation of the cathode material, reduced cycling stability, and an overall decline in battery performance. Furthermore, the dissolution of active materials contributes to capacity loss and inefficient cycling. To address these challenges, researchers have focused on developing protective coatings or surface modifications for the cathode materials. These coatings aim to prevent direct contact between the active material and the electrolyte, thereby minimizing undesirable reactions and improving cathode stability. Another approach involves the development of new electrolytes that are compatible with fluorine compounds. These electrolytes should exhibit excellent stability and avoid reacting with the cathode material, ensuring long-term performance and reliability. In conclusion, while cathode materials based on fluorine and chlorine compounds hold promise for high energy density batteries, their reactivity with the electrolyte and other components presents significant challenges. Researchers are actively exploring protective coatings and compatible electrolytes as potential solutions to enhance the stability and performance of these cathode materials [73,74].

**Figure 3:**
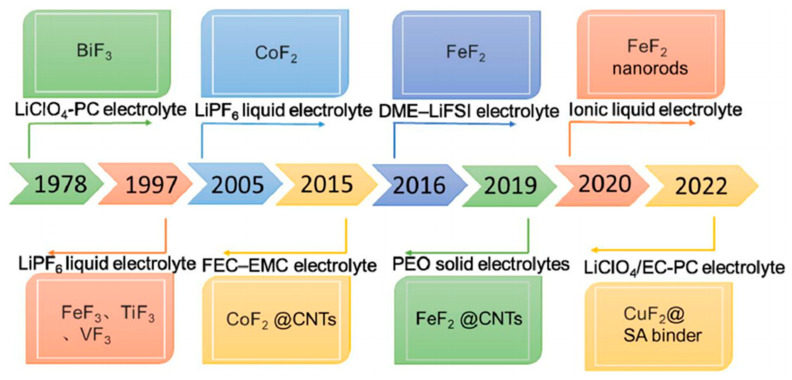
Development of MFx cathodes for Li-ion batteries over time, adapted from ref. [77], Wiley & Sons.

This review paper focused on nano scaling strategies to overcome the poor electrical and ionic conductivity of compounds like metal fluorides (MFx), such as FeF_3_, FeF_2_ (iron fluorides), CuF_2_, and CoF_3_. This conductivity issue arises because the metal–halogen bond in these compounds has a high ionic character as mentioned in the earlier section, resulting in a large band gap. Consequently, metal fluorides display limited electrical conductivity and ionic mobility. This limitation can be overcome by a combined approach of nanostructuring and hybridizing with conductive carbon or polymers [26,73,74]. Furthermore, cathode materials of type A undergo the formation of metal nanoparticles when fully lithiated. Metal nanoparticles catalyzed the electrolyte decomposition process. For instance, compounds like FeF_2_ and BiF_3_ have been observed to catalyze the decomposition of cyclic carbonates at higher voltages, resulting in reduced cyclic stability [75,76]. To address all these issues, various strategies such as ball milling, particle engineering, doping, and modifications of electrolytes have been investigated to stabilize reaction interfaces and mitigate the loss and degradation of active materials. The development strategies of MF_X_ performance are shown in Figure 3 [77].

### 3.1. Iron Trifluoride (FeF_3_)

Iron trifluoride (FeF_3_) is considered the most promising conversion cathode material, apart from FeF_2_. It exhibits a high theoretical capacity of 712 mA h g^−1^, offering significant potential to double the energy density of conventional commercial cathodes [15]. FeF_3_, CuF_2_, and CoF_3_ exhibit significantly smaller volume expansions of 14.06%, 11.54%, and 19.69%, respectively, in contrast to the substantial volume expansion of FeS_2_ (~159.20%) required to accommodate Li [30].

**Figure 4:**
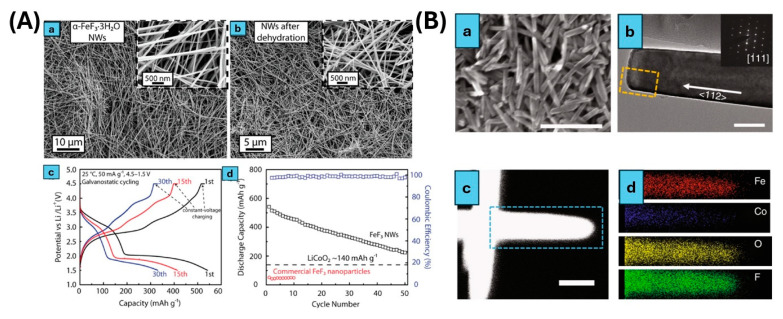
(**A**) SEM images of the α-FeF_3_·3H_2_O precursor (**a**) before and (**b**) after dehydration; FeF_3_ nanowire electrode: (**c**) voltage profiles, and (**d**) cycling performance. Reproduced with permission from ref. [78], American Chemical Society. (**B**) Fe_0.9_Co_0.1_OF nanorods: (**a**) SEM image, (**b**) TEM image, (**c**) STEM-HAADF image, where the rectangle denotes the elemental mapping area and (**d**) STEM-HAADF elemental mapping, adapted from ref. [66], Nature Publication.

A novel FeF_3_ nanowire (NW) synthesis through temperature-controlled dehydration in an inert atmosphere was reported by L. Li et al. [78]. SEM images revealed that NWs morphology preserved before and after dehydration with about 30–180 nm in diameter and 2–15 μm in length, as shown in Figure 4A(a,b). The FeF_3_ NW cathodes yielded a discharge capacity as high as 543 mA h g^−1^ at the first cycle but experienced capacity fading drastically and retained a capacity of 223 mA h g^−1^ after 50 cycles, as shown in Figure 4A(c,d). On a related note, Fan et al. used the solvothermal method to prepare co-doped iron fluoride (Fe_0.9_Co_0.1_OF) nanorods [66]. A uniform nanorod morphology of Fe_0.9_Co_0.1_OF with a diameter of about 40–50 nm was obtained where four elements (Fe, Co, O, and F) were uniformly distributed, as shown in Figure 4B. Stable electrochemical performance was demonstrated by this co-doped iron fluoride nanorods cathode, as displayed in Figure 2.

The micron-sized conversion-type FeF_3_·0.33H_2_O cathode offered a capacity of only 445 mA h g^−1^ [79]. An FeF_3_·0.33H_2_O nanoparticles (~50 nm) cathode displayed a capacity of only 438.9 mA h g^−1^ at the current density of 20 mA g^−1^ [80]. In contrast, a FeF_3_ nanocrystals composite with carbon showed a three-electron redox capacity (~700 mA h·g^−1^) up to 10 cycles at a considerably high rate of 500 mA·g^−1^. Chemically synthesizing FeF_3_ nanocrystals, and subsequent ball-milling with graphite, were the first two steps in producing FeF_3_/C nanocomposites [81].

**Figure 5:**
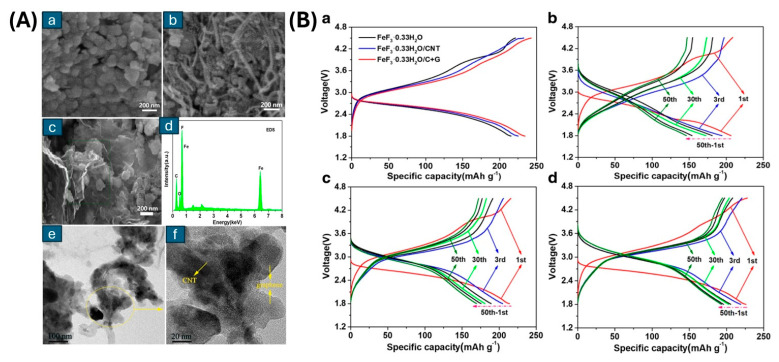
(**A**) SEM images of (**a**) FeF_3_·0.33H_2_O, (**b**) FeF_3_·0.33H_2_O/CNT, and (**c**) FeF_3_·0.33H_2_O/C + graphene; (**d**) EDS spectrum and (**e**,**f**) TEM images of the FeF_3_·0.33H_2_O/C+ graphene samples. (**B**) Electrochemical performance: (**a**) initial charge/discharge cycles of three electrodes and cycling performance of (**b**) FeF_3_·0.33H_2_O, (**c**) FeF_3_·0.33H_2_O/CNT, and (**d**) FeF_3_·0.33H_2_O/C + graphene electrodes, adapted from ref. [83], Springer.

Through an innovative approach, the use of porous carbon effectively prevented the growth of iron fluoride grains, accommodated volume changes in the active material, and facilitated electron or hole transfer to the electrochemical reaction sites, even in cases where certain regions of the active material were electrically isolated. This nano-confinement technique significantly reduced the dissolution of the iron fluoride cathode during cycling [82]. However, the cathode capacity (162 mA h g^−1^) substantially reduced due to excessive carbon mass. Lu et al. employed a liquid-phase technique to create a composite material consisting of FeF_3_∙0.33H_2_O embedded within a conductive network of carbon nanotubes (CNT) and graphene. Morphology analysis using SEM and TEM techniques revealed that FeF_3·_0.33H_2_O particles and CNTs and graphene sheets are closely interconnected with each other, as shown in Figure 5A. This approach was aimed at enhancing the electrochemical capabilities of the FeF_3_∙0.33H_2_O cathode material (Figure 5B) [83].

### 3.2. Iron Fluoride (FeF_2_)

FeF_2_ has an impressive theoretical capacity of 571 mA h g^−1^, attributed to its ability to involve two electrons per transition metal in the electrochemical process (FeF_2_ + 2Li^+^ + 2e^−^ → Fe + 2LiF). Additionally, FeF_2_ offers a high theoretical voltage (2.66 V vs. Li/Li^+^) due to the high electronegativity of the fluorine atom. Consequently, FeF_2_ boasts an extraordinary theoretical energy density, reaching 1519 Wh kg^−1^ [68,84,85].

A novel sugar-assisted solvothermal technique is employed to produce FeF_2_ nanomaterial, utilizing FeF_3_·3H_2_O as the precursor. The process transforms the precursor into FeF_3_∙0.33H_2_O nanoparticles, subsequently reduced into FeF_2_ nanoparticles by carbon derived from sugar dehydration and condensation. The resulting irregular granules are 30 nm with inner pores, and the first delivered discharge specific capacity was 561.7 mA h g^−1^. The capacity declined rapidly and remained only 40 mA h g^−1^ after 50 cycles in a carbonate-based electrolyte [86]. This outcome suggests that FeF_2_ electrode needs electrolyte optimization in addition to nano-structuring to become a potential cathode material for LIBs. M. Pasta and co-workers successfully fabricated FeF_2_ nanorods in a chemical synthesis process, which demonstrated a very high capacity (570 mA h g^−1^) and extraordinary cycling stability (>90% capacity retention after 50 cycles at C/20) using an ionic liquid electrolyte (1 m LiFSI/Pyr1,3FSI) [87].

**Figure 6:**
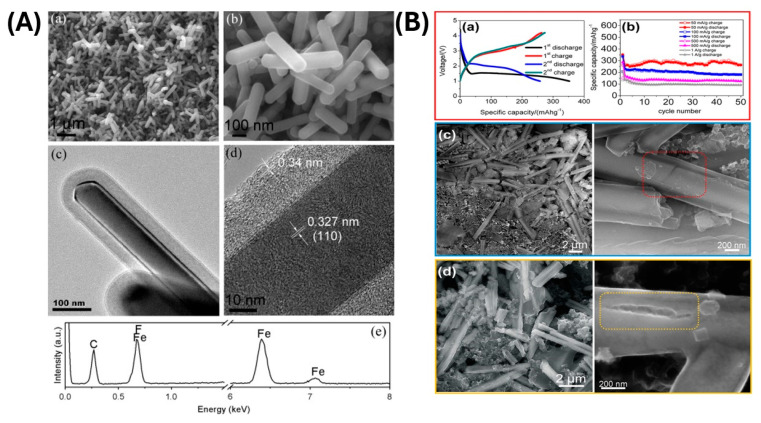
(**A**) FeF_2_–carbon core–shell composite: (**a**,**b**) SEM images; (**c**,**d**) TEM images, and (**e**) EDS spectrum, reproduced with permission from ref. [88], Elsevier. (**B**) FeF_2_@CNT cathode (**a**) two initial charge/discharge profiles and (**b**) cycling performance at different current densities and SEM images (**c**) before and (**d**) after 50 charge/discharge cycles, reproduced with permission from ref. [89], American Chemical Society.

He and his coworkers’ work [88] demonstrated a one-pot method to synthesize FeF_2_–carbon core–shell nanorods. The carbon shell thickness was 10–20 nm, and the nanorod diameters ranged from 100 nm to 1 m, as shown in Figure 6A. The FeF_2_@C nanorod cathode exhibited an initial reversible capacity of 314 mA h g^−1^ and maintained cycle retention up to 50 cycles with a fade rate of 0.74%. Zhou et al. encapsulated FeF_2_ nanorods into a carbon nanotube to generate a stable cycling performance. The electrode exhibited a reversible capacity of 263 mA h g^−1^, which was retained up to 50 cycles with no capacity fading, as shown in Figure 6B(b) [89]. FeF_2_@CNTs maintain one-dimensional nanorod morphology after 50 cycles, as presented in Figure 6B(c,d). A magnified SEM image (yellow box in Figure 6B) shows that the carbon shell cracked after 50 cycles, which indicates that high internal stress occurred during the Li-ion insertion process.

Crystalline FeF_2_ nanoparticles (~35 nm) covered with a thin carbon layer (~2–3 nm) (FeF_2_@C) were synthesized via microwave irradiation and demonstrated excellent performance as a cathode in LIBs [90]. FeF_2_@C delivers a capacity of 634 mA h g^−1^ after 50 cycles at C/20, whereas a similarly prepared commercial FeF_2_ material only provides 234 mA h g^−1^. A pressure-induced morphological control technique has recently been utilized to synthesize coralloid-like FeF_2_ nanocrystals with a nitrogen-enriched carbon coating, referred to as c-FeF_2_@NC. This structure reduces interfacial resistance and enhances topotactic transformation during conversion reactions. The coating enhances interfacial stability and kinetic performance. When utilized as a conversion cathode for LIBs, c-FeF_2_@NC demonstrates a high initial reversible capacity of 503.57 mA h g^−1^ and exceptional cycling stability, maintaining 497.61 mA h g^−1^ with a minimal capacity decay of 1.19% over 50 cycles [91]. In addition, a method was developed in a recent study to passivate the cathode/electrolyte interface by embedding FeF_2_ nanoparticles in a cyclic polyacrylonitrile (cPAN) network. The cPAN acts as a strong binder, preventing excessive CEI growth and achieving stable cycling in a lean electrolyte. This binder-free FeF_2_-cPAN cathode is paired with a high-capacity SiO/C anode, exhibiting a capacity of 400 mA h/g after 100 cycles at 0.5 C [92].

### 3.3. Cupper Fluoride (CuF_2_)

CuF_2_ is a prominent candidate among MFx cathode materials due to its high theoretical voltage of 3.55 V and impressive energy densities, 1874 Wh kg^−1^ gravimetric and 7870 Wh L^−1^ volumetric. Despite these promising characteristics, its strong ionic bonds result in poor electronic conductivity, hindering its electrochemical effectiveness. Additionally, CuF_2_’s hygroscopic nature means it absorbs moisture, leading to impurities such as CuF_2_·H_2_O and CuOHF that are hard to remove with heat treatment and negatively impact performance [93]. To address these issues, researchers have focused on understanding the reaction mechanisms between CuF_2_ and lithium during charge and discharge cycles, with the goal of enhancing its electrochemical performance [94]. In 2016, researchers developed a reversible CuF_2_ electrode coated with NiO, which significantly enhanced the material’s reversibility by preventing direct contact between copper nanoparticles and the electrolyte. It was discovered that the size of the metal nanoparticles plays a critical role in determining the voltage of the conversion reaction. The surface energy of these nanoparticles adversely affects the free energy of the conversion reaction, leading to a reduction in the observed voltage compared to the theoretical value. This finding highlights the inherent challenges in achieving complete electrochemical reversibility for CuF_2_ [95]. Recent research has demonstrated that porous anhydrous CuF_2_ with a micro-nano-hierarchical structure is an effective cathode material for LIBs [96]. The porous CuF_2_ demonstrates an exceptional initial discharge capacity of 523 mA h g^−1^ (0.1 C) and a remarkable rate capacity of 403 mA h g^−1^ (5 C), with a cutoff voltage of 1.5 V versus Li/Li^+^. This innovative material exhibits exceptional electrochemical performance due to its unique structure, which facilitates rapid ion transport and enhances the charge storage capacity. Consequently, it holds significant promises for improving the energy density and longevity of LIBs. In addition, a pomegranate-Like CuF_2_ cathode derived from spent lithium-ion batteries exhibits a large specific capacity (~535 mA h g^−1^ at 50 mA g^−1^) and superb cycling stability (at 70 cycles, it retains 100% of its initial capacity) [97]. In this approach, a peripheral SEI layer was introduced via solvothermal heating of a three-dimensional Cu foam in a spent LIB electrolyte (LiPF_6_) solvent to accommodate the large volume expansion of CuF_2_ during electrochemical cycling.

### 3.4. Cobalt Fluoride

Cobalt fluorides, particularly CoF_3_ and CoF_2_, are notable in battery technology. CoF_3_ is often used in primary batteries, but its application as a cathode in lithium-ion batteries is complicated by issues such as electrolyte decomposition. CoF_2_, on the other hand, is promising due to its theoretical capacities 553 mA h g^−1^ gravimetric and 2038 mA h cm^−3^ volumetric along with a theoretical voltage of 2.854 V and an energy density of 1578 Wh kg^−1^ [98]. Its major challenge, however, is low electronic and ionic conductivity, which affects its cycling performance negatively.

Conductive carbon additives are considered essential for enhancing the capacity retention of CoF_2_ materials. Researchers successfully created a CoF_2__CF composite electrode using a straightforward hydrothermal process. The resulting composite demonstrated a high specific capacity, impressive rate capability, and strong cycling performance [98]. The electrochemical performance of electrode materials can be greatly influenced by factors such as the morphological structure, crystallinity, solvothermal temperature, and reaction time. In a particular study, CoF_2_ spheres were synthesized to possess a porous structure and high crystallinity, resulting in notable electrochemical performance. CoF_2_ encapsulated in an interconnected N-doped carbon matrix exhibited a notable reversible capacity of 352.0 mA h·g^−1^ at 200 mA·g^−1^ [99]. Though the specific capacity somewhat reduced due to excessive carbon mass, superior long-term cycling stability (1200 cycles) even at a high current density of 2000 mA h g^−1^, was demonstrated. This study introduces innovative synthesis approaches for designing high-energy metal fluoride materials. These approaches involve novel chemical reactions and advanced fabrication techniques, leading to materials with enhanced energy storage capabilities. The resulting high-energy metal fluorides have potential applications in battery technology and other energy-related fields. In addition, a CoF_2_/multi-walled carbon nanotube (MWCNT) nanocomposite cathode with improved reversible capacities (554 mA h g^−1^ at 0.2 C) and superior rate capabilities (a capacity retention of 84.7% at 2 C versus 0.2 C) was also reported as a potential cathode material in a recent study. This nanocomposite was developed using a facile precipitation method [100].

In the latest study, a CoF_2_@C cathode material with MOF structure was inspected to establish a relationship between the cathode electrolyte interface (CEI) failure mechanism and electrochemical cycling [101]. The FEC-based electrolyte induced LiF-rich and uniform CEI, stimulating faster Li⁺ ion kinetics and offering a high and stable capacity at 0.1 C, even after 300 cycles. This stable performance indicates the potential of FEC-based electrolytes in enhancing the longevity and efficiency of lithium-ion batteries, making them more reliable for long-term use in various applications. Investigations into CoF_2_ thin films within Li/LiPF_6_/Lipon/CoF_2_ battery systems, focusing on its performance in the 0.01–3.5 V range, have provided a basis for further research on its use in energy storage applications [102].

### 3.5. Other Metal Fluorides

**Figure 7:**
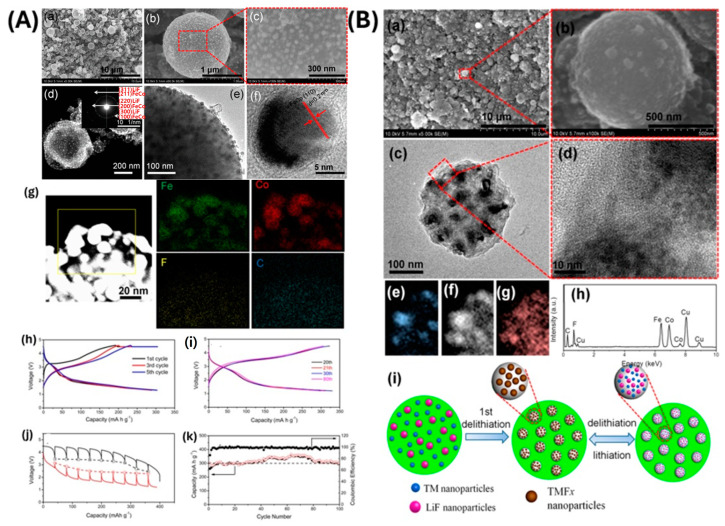
(**A**) FeCo/LiF/C nanocomposites: (**a**–**c**) SEM images; (**d**) HAADF-STEM image with SAED patterns; (**e**) TEM image; (**f**) HRTEM image of the dark nanoparticle in (**e**); (**g**) elemental distribution of Fe, Co, F, and C determined by EDS mapping; (**h**) charge/discharge profiles of initial five cycles, (**i**) charge/discharge profiles for different cycles (**j**) quasi-equilibrium voltage profile obtained from galvanostatic intermittent titration technique measurements, (**k**) cycling performance at 30 mA g^−1^. (**B**) SEM (**a**,**b**), TEM (**c**), and HRTEM (**d**) images of the pomegranate-like nanocomposite electrode charged to 4.5 V after the fifth cycle, then kept at 4.4 V for 48 h. (**e**–**g**) Electron energy loss spectra of Co, Fe, and F recorded for the area shown in (**c**). (**h**) Corresponding EDS results of (**c**). (**i**) A schematic representation of the impact of cycling on the microstructural evolution of the FeCo/LiF/C sphere electrode. Reproduced with permission from ref. [70], American Chemical Society.

A nanocomposite containing FeM/LiF/C (M = Co, Ni) with a pomegranate-structured morphology was studied [70], where primary LiF nanoparticles and FeM nanoparticles coated with uniform graphene layers (2–3 nm in thickness) are arranged as distinct pomegranate-like seeds within a carbonaceous secondary microsphere (100–1000 nm), as depicted in Figure 7A(a–f). This unique structure enhances the electrochemical performance of the nanocomposite, providing a high capacity and excellent cycling stability. The uniform graphene coating facilitates efficient electron transport, while the carbonaceous microsphere prevents the aggregation of the nanoparticles. EDS mapping shows homogeneous F and C elements within spheres, as presented in Figure 7A(g), implying LiF integration into a carbon composite matrix. Fe and Co mapping overlap confirms alloying, and FeCo nano crystallites are evenly dispersed in LiF and carbon vicinities, indicating intimate contact among all components. The as-synthesized ternary nanocomposite FeCo/LiF/C exhibits reversibility with a high capacity of ~300 mA h g^−1^ at 1.2–4.5 V for over 100 cycles, as shown in Figure 7A(h–k). After the fifth cycle, the composite maintained a pomegranate-like architecture (Figure 7B(a–d)), while the electron energy loss spectra revealed a detailed structure and elemental distribution, as shown in Figure 7B(e–g). The nanocomposite electrode showed a distribution of Fe, Co, and F, with most F overlapping with Fe or Co areas. The proposed conversion–reaction mechanism of the nanocomposite was derived from the electrochemical behavior and the structural/morphological changes observed in Figure 7B(i).

A noble approach involving dual metal accelerated LiF splitting was used to produce a composite cathode (LiF/Fe/Cu) with superior conversion reaction properties [103]. Various molar ratios of LiF, Fe, and Cu powders were mixed and pressed at a pressure of 4 MPa cm^–2^ to form the targets. A pulse laser deposition (PLD) method was used to grow LiF/Fe/Cu, LiF/Fe, and LiF/Cu thin film electrodes on Pt-Ti-covered Si substrates. With this approach, significant reversibility is achieved with a capacity of 375–400 mA h g^−1^, which has high-capacity retention (at least 200 cycles). This innovative approach offers a promising strategy for developing high-performance conversion cathode materials for advanced lithium-ion batteries.

## 4. Sulphur and Sulfide Compounds

**Figure 8:**
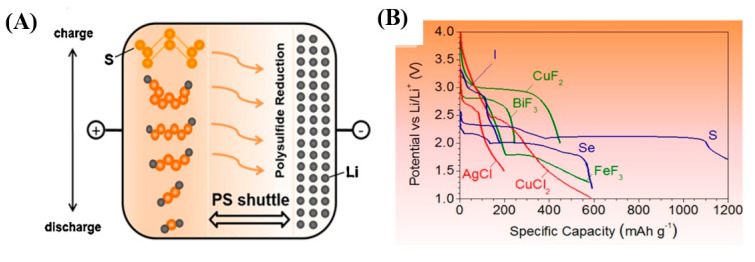
(**A**) Polysulfides migrate during the charging and discharging process of an S particle. (**B**) Representative discharge curves of various conversion cathodes, adapted with authorization from reference [9], Elsevier.

Sulfur (S) has gained significant attention as a widely studied cathode material, which is an attractive choice due to its advantageous properties, affordability, and abundant availability on Earth. Figure 8A illustrates the intermediate steps involved in the complete conversion reaction of S, where polysulfide intermediates are solubilized in an organic electrolyte, which is a substantial disadvantage of sulfur cathode [104]. On the other hand, Figure 8B depicts the discharge curves of various conversion cathodes, including S-, Se-, and I-based compounds, CuCl_2_, AgCl, BiF_3_, and CuF_2_. Among these cathodes, BiF_3_ and CuF_2_ demonstrate favorable discharge characteristics featuring elevated voltage plateaus. In contrast, S- and Se-based cathodes exhibit voltage plateaus that are relatively flat and extended, indicating good reaction kinetics between the two solid phases [9].

Sulfur and metal sulfide nanostructures are promising in the field of rechargeable batteries due to their potential to enhance performance metrics such as energy density, cycle life, and rate capability. These materials show significant promise as cathodes in lithium–sulfur (Li-S) batteries, which are considered cutting-edge energy storage technology. Lithium–sulfur batteries (LSBs) facilitate energy storage and transfer through reversible electrochemical interactions between lithium and sulfur, which enable LSB cathodes, with elemental sulfur offering high theoretical energy density reaching up to 2600 Wh kg^−1^, surpassing that of commercial lithium-ion batteries (LIBs) by fivefold. Additionally, its theoretical specific capacity of 1675 mA h g^−1^ presents a significant advantage over conventional cathode materials [105]. However, its practical application is hindered by challenges such as the dissolution of polysulfides (Li_2_S, Li_4_S_8_, etc.) into the electrolyte, which leads to the notorious shuttling effect and a rapid capacity fade [106]. As a result, insoluble polysulfides move to the sulfur cathode, where they are converted back into long-chain lithium polysulfides. This is called the “shuttle effect”, where polysulfides dissolve and migrate between the cathode and anode, causing self-discharge, capacity fading, and poor cycling stability. This conversion reduces the number of active materials, which adversely affects both the cycle life of LSBs and their coulombic efficiency. Additionally, this process leads to significant volumetric changes in the sulfur cathode during charging and discharging. The densities of sulfur (S) and lithium sulfide (Li_2_S), the discharge product, are 2.03 g/cm^3^ and 1.66 g/cm^3^, respectively. This difference means the sulfur cathode can expand or contract by as much as 80% during electrochemical reactions [107].

To address challenges in lithium–sulfur batteries, researchers have explored various electrolyte formulations and composite cathode materials. The use of solid and gel-like electrolytes, along with lithium nitrate (LiNO_3_) to reduce the dissolution of lithium polysulfides (LiPSs), has been shown to enhance cycling performance [108]. Additionally, modifying LSB cathodes via nano scaling and combining sulfur with conductive matrix materials has aimed to improve key electrochemical metrics, including specific capacity, cycling stability, and conductivity. Nano structuring is crucial for sulfur cathode performance, and vinylene-linked cationic COFs, with their electrostatic polarity controlled by varying counter anions, can serve as ionic sieve membranes to further enhance battery performance [109]. Nano structuring is indeed crucial for sulfur cathode performance improvement. Here is how nano structuring can help:

- Polysulfide Confinement

Nano structuring can help to confine these polysulfides within the cathode structure. By using materials that have enough surface area and porous structures (such as carbon-based nanomaterials), it is possible to trap polysulfides and reduce their migration, thus minimizing the shuttle effect. A smaller particle size and broader surface-to-volume ratio of the nanostructures can significantly enhance the interaction between the sulfur and the matrix material, increasing the capacity retention and overall performance. Moreover, nano structuring often offers dual confinement—physically trapping polysulfides in their porous architecture and chemically binding them through functional groups or dopants. This dual approach stabilizes sulfur chemistry, ensuring structural integrity and a longer cycle life with better capacity retention [110].

- Enhanced Conductivity

Nanostructured materials, especially conductive nanomaterials like carbon nanotubes (CNTs), graphene, and other conductive polymers, can deliver better electron direction within the sulfur cathode. This lowers the rate of charge transfer resistance and enhances the overall electrochemical performance. For example, carbon nanostructures (e.g., carbon nanotubes, mesoporous carbon) embedded within the sulfur cathode act as conductive frameworks, improving the conductivity of the sulfur cathode and promoting better electron flow during charge/discharge cycles.

- Increased Surface Area and Porosity

Nanostructured materials like porous carbons, mesoporous silica, or carbon–sulfur composites increase the surface area and porosity within the cathode. This offers reactive surface for sulfur and lithium ions to interact, which can enhance the overall capacity of the cathode. More importantly, the porous network can host the polysulfides, minimizing their loss to the electrolyte and thus upgrading the cycling performance and durability of the battery.

Moreover, the nano structuring of metal sulfides and sulfur can significantly accommodate the volumetric changes that occur during cycling [111]. By reducing the material to the manometer scale, the surface-to-volume ratio is increased, allowing for better electrolyte penetration and enhanced sulfur and electrolyte interaction. The use of nanocomposites, where sulfur is embedded into conductive materials or mixed with a host material, offers better mechanical support and chemical stability. For instance, sulfur can be confined inside porous carbon structures, which helps with both polysulfide confinement and enhanced conductivity.

Examples of nanostructures used include the following:

- Carbon Nanotubes (CNTs): These can be utilized as conductive cathode additives to develop a network that enhances the overall performance and helps in polysulfide confinement.
- Graphene: The 2D structural properties of graphene offer a wide surface area and high conductivity, which can trap polysulfides effectively and also provide efficient electron pathways.
- Mesoporous Carbon: These materials have a large surface area and can offer a host for sulfur, helping to confine polysulfides and protect them from dissolving in electrolytes.
- Metal–Organic Frameworks (MOFs): These can offer a highly porous structure and have the potential to adsorb and confine polysulfides.

Incorporating nanostructured metal sulfides in a sulfur cathode involves several methods, such as physical mixing, chemical vapor deposition, or in situ synthesis, each capable of tailoring the architecture to optimize performance characteristics. These designs often lead to hierarchical structures that enhance electron and ion transport, increase sulfur utilization, and stabilize the overall structure of the cathode. Research continues to explore various metal sulfide composites, surface modifications, and hybrid materials to overcome current limitations and push the boundaries of what sulfur-based cathodes can achieve [112]. With ongoing advancements, sulfur and metal sulfide nanostructure cathodes hold great promise for creating more efficient, durable, and sustainable lithium-ion batteries, potentially transforming the energy storage landscape. Therefore, herein we review different sulfur-containing composite cathode materials fabricated with carbon-derived nanomaterials.

### 4.1. Sulfur and Carbon-Based Composites

Carbon materials are widely utilized as sulfur carriers in LSBs due to their low density, excellent conductivity, and stable electrochemical properties, and their superior mechanical strength enables the creation of flexible electrodes [113,114,115]. Additionally, carbon nanomaterials show wide surface and pore volumes, enhancing sulfur loading and improving the kinetics at the electrode–electrolyte interface. Their adsorption capabilities also help prevent the diffusion of lithium polysulfides (LiPSs), effectively reducing the shuttle effect and enhancing overall electrochemical performance. Common carbon structures used to modify sulfur cathodes include graphene, CNTs, porous carbon, and hollow carbon due to their unique morphologies and functionalities. The robust free-standing 3D architecture of interlaced carbon nanofibers (CNFs) provides significant structural and mechanical strength during volume changes at high sulfur loadings [116,117]. Typically, activation processes or sacrificial templates are employed to create these CNF structures. Moreover, doping CNFs with heteroatoms presents an effective method for enhancing their performance in Li-S batteries. This chemical doping not only optimizes textural properties—such as porosity and surface area—but also improves electrical conductivity, facilitates ionic transport, and creates anchoring sites for strong interactions with intermediate polysulfides [118,119].

For instance, a novel cathode was designed to boost Li–S pouch cell performance through advanced nanostructures. The cathode integrates ZnS nanoparticles and a Co–N–C single-atom catalyst, forming dual-end binding sites within a structured macro-porous framework. This configuration stabilizes and catalyzes polysulfide intermediates during cycling, mitigating the corrosion of the lithium anode and reducing the shuttle effect. The structured macropores improve ionic transport under high sulfur loading by creating triple-phase boundaries between the catalyst, conductive matrix, and electrolyte, thereby minimizing the development of dead sulfur. Consequently, the cathode shows superior performance, even with high sulfur content and lean electrolyte. The rate capability test indicates that the 3d-omsh/ZnS, Co–N–C/S cathode achieves the maximum specific capacity across various rates (0.15 C to 5.0 C) [120].

**Figure 9:**
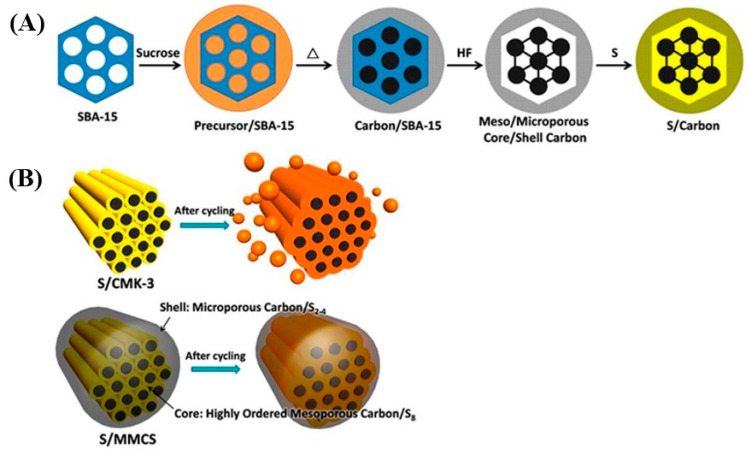
(**A**) Synthesis schematic of highly ordered (MMCS) carbon and sulfur and carbon composite. (**B**) Explanation of the mesoC/S_8_-microC/S_24_ core shell structure, reproduced with permission from ref. [121]. American Chemical Society.

Li et al. [121] developed an ordered meso-microporous core–shell (MMCS) carbon structure to serve as a sulfur container (as shown in Figure 9). The core’s large pore volume and organized porous design allow for substantial sulfur loading and the effective utilization of active materials. The shell, made of microporous carbon and smaller sulfur particles, acts as a barrier to enhance cycling stability. This composite achieves a capacity of 837 mA h g^−1^ at a rate of 835 mA g^−1^ after 200 cycles, maintaining 80% of its capacity compared to the second cycle. Additionally, this bimodal structure significantly reduces polysulfide diffusion into the bulk electrolyte.

**Figure 10:**
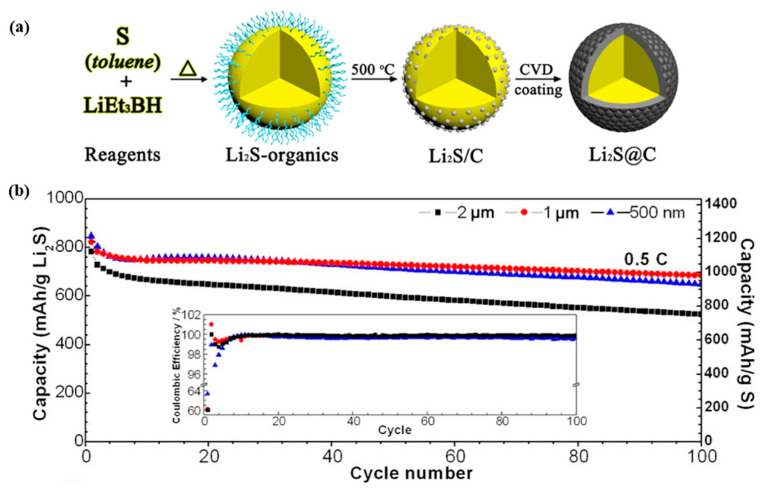
(**a**) Diagram illustrating the synthesis and coating process of Li_2_S@C spheres. (**b**) Cycling performance comparison of 2 μm, 1 μm, and 500 nm Li_2_S@C at a 0.5 C rate, reproduced with permission from ref. [122]. American Chemical Society.

C. Nan et al. [122] pioneered the synthesis of size-controlled Li_2_S spheres and successfully utilized a chemical vapor deposition method to create stable core–shell particles composed of Li_2_S coated with carbon (Li_2_S@C). These Li_2_S@C particles, featuring protective and conductive carbon layers, exhibit impressive electrochemical performance, achieving an initial discharge capacity of 972 mA h g⁻^1^ at a 0.2 C rate with stable cycling performance and little morphological change even after 400 cycles at a 0.5 C rate, despite lacking additional carbon reinforcement (as shown in Figure 10).

### 4.2. Sulfur and Graphene Composites

Graphene oxide was stirred with polysulfides under a lower pH, then oxidized in situ, filtered, and dried to create a graphene–sulfur composite. Morphological characterization showed that sulfur microparticles were totally encased in graphene sheets, forming a conductive network that effectively captured polysulfides through hydrophilic interactions. The authors of [123] designed a graphene sponge-based cathode with a high sulfur capacity for bendable applications of lithium–sulfur batteries, synthesized via chemical vapor deposition on nickel foam and then coated with polydimethylsiloxane.

**Figure 11:**
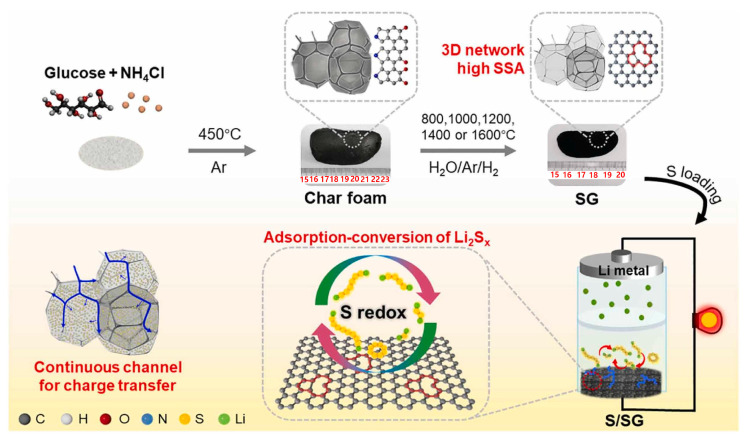
Fabrication of S/SG and its role on redox behavior of Li-S batteries [124].

In recent advancements in Li-S battery technology, Zhou et al. [124] developed a strutted graphene (SG) foam, which serves as an effective sulfur host (Figure 11). This foam is characterized by interlinked networks, porous frameworks, and persistent imperfections, which collectively enhance the cathode’s conductivity, accommodate volumetric changes, and promote efficient electron and ion transport. Moreover, these features help mitigate the shuttle effect, leading to significant improvements in battery performance. In a complementary approach, researchers employed a synergistic strategy by utilizing a nitrogen-doped three-dimensional graphene aerogel. This material serves dual roles: as a host for the lithium anode and to ensure uniform lithium plating and stripping, thereby reducing dendrite formation; as a host for the sulfur cathode, it enhances sulfur redox reactions and tackles the polysulfide shuttling effect, ultimately achieving a high energy density and prolonged cycle life [125]. These advancements depict a promising future for Li-S batteries by addressing crucial challenges through innovative material design.

### 4.3. Sulfur and CNT Composites

Razzaq et al. developed a binder-free freestanding thin-film composite cathode for lithium–sulfur batteries (LSBs) composed of sulfurized polyacrylonitrile (PAN) and carbon nanotubes (CNTs) (SPCN–CNT). This composite was fabricated through the co-electrospinning of a ternary precursor blend of sulfur, PAN, and CNTs, followed by vulcanization at elevated temperatures. The interplay between sulfur and CNTs promoted the formation of a porous nanofibrous structure, which substantially improved the electrochemical performance of the cathode [126].

Wu et al. proposed a polymeric sulfur/CNT composite featuring 2,5-dithiobiurea (DTB) to enhance the stability of long-chain sulfur. The process involved synthesizing polymeric sulfur via a ring-opening reaction, leading to the creation of sulfur-DTB (S-DTB) through interactions at the terminal ends of the sulfur polymer chain. CNTs, functionalized with carboxylic acid groups and DTB, were then coupled with S-DTB through an amide reaction, resulting in a coaxial composite, designated SDCD [127]. Zheng et al. utilized ultrasonic-assisted repeated wet impregnation and simultaneous drying techniques to embed copper nanoparticles within a microporous carbon matrix, producing a copper-stabilized sulfur microporous carbon composite [128]. The method ensured a uniform dispersion of Cu nanoparticles before sulfur loading via wet impregnation to the cycle stability and rate performance of LSB cathodes by electrochemically impregnating sulfur into hierarchical mesoporous carbon nanoparticles, offering a viable approach for improving battery efficiency and longevity [129].

**Figure 12:**
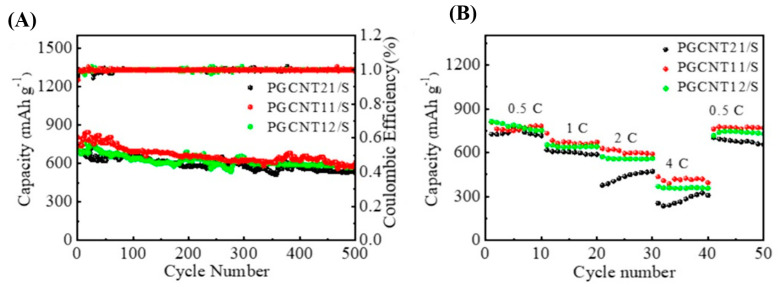
The electrochemical performance of different proportions of PGCNT/S electrode in LiS battery. (**A**) Long cycle curve and coulombic efficiency at current density of 1 C. (**B**) Rate performance [130].

A study synthesized a recrystallized g-C_3_N_3_ (PGCN)/CNT composite using g-C_3_N_3_ and carbon nanotubes via a dissolution–precipitation method to enhance Li-S battery performance [130]. An optimal 1:1 mass ratio of PGCN to CNTs was found to improve electrochemical properties significantly. The interconnected CNT network adsorbs lithium polysulfides (LiPSs) physically, The pyridinic nitrogen sites on PGCN nanosheets promote chemical adsorption. The PGCNT11/S electrode delivered an initial discharge specific capacity of 803.4 mA h g⁻^1^ at 1 C and retained 605.0 mA h g⁻^1^ after 500 cycles, demonstrating its ability to reduce the polysulfide shuttle effect and enhance battery stability (Figure 12).

### 4.4. Sulfur and Metallic Oxide Composites

To improve the tap density of lithium–sulfur battery (LSB) cathodes, polar inorganic nano compounds, particularly transition metal oxides, are commonly employed as sulfur hosts [131]. These metallic oxides, characterized by oxidized oxygen anions on their surfaces, exhibit strong polarity, which effectively absorbs the polysulfides generated during electrochemical reactions [132]. The synthesis of sulfur/manganese dioxide (S@MnO_2_) hybrid materials was performed by encapsulating sulfur nanospheres within α-MnO_2_ shells using an environmentally amiable strategy that can be performed at room temperature [133]. The resulting electrode demonstrated a capacity of 760 mA h g^−1^ after 200 cycles at a current density of 0.1 A g^−1^, with sulfur content maintained at 72.5 wt.%. This energy-efficient and time-saving fabrication method demonstrates strong potential for the future application of this hybrid sulfur cathode. Li et al. [134] developed an enhanced sulfur cathode material featuring a core–shell configuration, with sulfur nanospheres in the inner layer and an ultrathin α-MnO_2_ nanosheet in the outer layer, produced through simple precipitation techniques. MnO_2_/S composites utilizing mesoporous MnO_2_ nanofibers (100–120 nm in diameter) as the host material were also explored [135]. The initial specific capacity of this cathode was 1306 mA h g⁻^1^ at 0.2 C, which dropped to 1205 mA h g⁻^1^ after 200 cycles, yielding a capacity retention of 89%. These results emphasize the promising design approaches for advancing energy storage applications in LSB cathodes. Moreover, the use of unique rutile TiO_2_ mesocrystals as a sulfur host in LSBs and a polyhedral TiO_2_ (poly-TiO_2_) with sulfur-based cathodes attained a high volumetric capacity of 1145 mA h cm⁻^3^, nearly 9.1 times higher than that of commercial TiO_2_ nanoparticle cathodes [136]. Encapsulated sulfur nanospheres within multilayer V/V_2_O_5_ nanoshells (S@V/V_2_O_5_) produced an LSB cathode with up to 93 wt.% sulfur content. The composite electrode, containing 87 wt.% sulfur, exhibited a high specific capacity of 1403 mA h g⁻^1^, an initial areal capacity of 4.4 mA h cm⁻^2^ at 0.2 C, and excellent cycling durability with a specific capacity of 819 mA h g^−1^ after 300 cycles at 0.5 C [137]. A honeycomb-spherical sulfur cathode host was developed by arranging hollow metallic and polar Co_9_S_8_ nanotubes in an ordered manner. The precursor, consisting of nanorods, was transformed into a Co_9_S_8_ hollow structure through sulfurization, and sulfur was incorporated into the structure via melt diffusion. This S@Co_9_S_8_ composite cathode demonstrated excellent cycling and rate stability, achieving reversible capacities of 1136, 1011, 893, 842, and 806 mA h g^−1^ at current densities of 0.2, 0.5, 1.0, 1.5, and 2.0 C, respectively [138].

### 4.5. Metal Oxide-Based Nanocomposite

Metal oxide-based nanomaterial cathodes have appeared as a promising solution for enhancing the performance of lithium–sulfur batteries. Their high conductivity improves electron transfer during electrochemical reactions, while their polar surfaces effectively adsorb lithium polysulfides (LiPSs), mitigating the notorious shuttle effect that contributes to capacity fading. Additionally, metal oxides provide structural stability to cathodes, managing the volume fluctuations linked to sulfur redox reactions. The tunable properties of metal oxide nanomaterials, including porosity and morphology, further enhance sulfur loading and reaction kinetics. The performance of various nano metal oxide cathodes for lithium–sulfur batteries is summarized in Table 1. In 2024, Bin Yue et al. [139] introduced an innovative structure called Co/CoN-carbon nanocages@TiO_2_-carbon nanotubes (NC@TiO_2_-CNTs) created via an electrospinning and nitridation process.

When used as cathode materials in lithium–sulfur batteries, the NC@TiO_2_-CNTs exhibited excellent sulfur utilization, achieving a discharge capacity of 1527 mA h g^−1^ at a current rate of 0.2 C [139].

## 5. A Realistic Look at Conversion Cathode

### 5.1. Scalability of the Nano Structured Conversion Cathode

While conversion cathodes hold significant potential for next-generation lithium-ion batteries because of their high theoretical capacity and energy density, their broader commercialization is still hindered by challenges like large volume changes during cycling, poor electrical conductivity, and issues managing the large interfacial area created during the conversion reaction; despite ongoing research efforts to address these limitations, they are not yet widely used in commercially available batteries [15]. However, these materials have the potential to be commercialized in the future.

Various synthesis techniques are utilized to produce conversion cathode materials, such as solid-state synthesis, ball milling, precipitation, hydrothermal/solvothermal, and template-assisted synthesis [28,154,155]. Among these, precipitation synthesis stands out because this method can be adapted to produce large quantities of material relatively quickly, which is advantageous for industries requiring high-volume production. Hydrothermal and solvothermal methods also offer controlled synthesis under specific conditions, although they require further optimization for broader scalability. Mechanical ball milling presents a lower-cost option with existing industrial applications, but uniformity and morphology control still need attention [156].

While nano-structuring strategies hold immense potential for improving the performance of conversion cathodes in energy storage devices, scaling these techniques for industrial applications is not without challenges. Large-scale production of nano-structured materials often results in energy-intensive processes, which can reduce the overall efficiency and sustainability of the manufacturing process. Nano-structured materials often show high batch-to-batch variability during synthesis, which can lead to inconsistent performance in the final products. Inconsistent material properties can affect the cycle life and overall efficiency of the batteries, which is a major concern for industrial applications. Many nano-structuring methods (such as ALD, vapor deposition, and sol-gel processes) can be expensive and complex to scale up [157,158]. For industrial manufacturing, processes need to be cost-effective, reproducible, and capable of handling large quantities of material. Techniques like atomic layer deposition are highly precise but come at a higher cost, making them less suitable for mass production without significant advancements in scalability. In the synthesis of conversion-type cathodes, the chemical vapor deposition (CVD) method can be employed to create nanostructured thin films, though this approach is typically more expensive and complex, necessitating specialized equipment compared to other methods.

The cost and complexity of synthesis, maintaining uniformity, and ensuring stability are key barriers. However, with ongoing research and development into more cost-effective, scalable synthesis methods, as well as improvements in material stability and manufacturing processes, nano-structuring could play a significant role in the next generation of high-performance batteries. Feasibility for large-scale industrial adoption will depend on advancements in process engineering, material innovation, and cost reduction. Finally, the large-scale adoption of nano-structured conversion cathode materials will depend on both the battery industry’s willingness to invest in new technologies and the establishment of standardized production processes. Over time, as the benefits of nano-structuring become more evident, companies may shift toward scaling these materials for mass production.

There are significant trade-offs between cost, performance, and manufacturability in selecting materials for conversion-type cathodes in lithium-ion batteries. State-of-the-art LIB cathode materials, which often contain rare or expensive transition metals (such as Ni and Co), can elevate manufacturing costs significantly, whereas more abundant materials may compromise performance in terms of energy density and lifespan. Metal fluoride derived from iron and copper can serve as a favorable compromise. Additionally, metal sulfide free of Ni and Co can be a favorable option. Moreover, advanced fabrication techniques that yield superior materials typically require expensive equipment and complex processes, which can limit scalability despite their high-quality output [19]. In contrast, simpler methods like sol-gel synthesis or mechanical milling may reduce costs and enhance manufacturability but might not achieve the same electrochemical performance. Additionally, safety and environmental considerations also play a crucial role, with materials that are toxic or challenging to manage potentially facing regulatory hurdles. Thus, manufacturers must navigate these trade-offs strategically to find the right balance that aligns with specific application requirements and market demands.

### 5.2. Long Time Cyclability of Conversion Cathode

The long-term cycling durability of conversion cathodes is a crucial element affecting their real-world use in lithium-ion and lithium–sulfur batteries. These cathodes, while capable of delivering high energy densities, often suffer from significant structural degradation and capacity loss during extended cycling due to large volume changes and mechanical stresses induced by the conversion reaction. This instability can lead to detrimental effects such as the dissolution of active materials, breaking electrical interaction, and increased internal resistance, ultimately undermining the battery’s overall efficiency and lifespan. To enhance cycling stability, it is urgent to develop the nanostructures of these cathodes, improve the mechanical properties of the electrode materials, and develop suitable conductive matrices that can accommodate volume fluctuations. Additionally, formulating advanced electrolytes that minimize side reactions and promote stability under cycling conditions is vital for achieving consistent performance over extended charge-discharge cycles. Mentioning these difficulties would be key to realizing the full potential of conversion cathodes in commercial battery applications.

The key limitation that restricts the application of conversion-type cathodes lies in their poor reversibility during cycling. When these cathodes, represented by the general formula MX_n_, react with lithium during the discharge phase, the atomic structure undergoes significant rearrangements. This leads to the formation of several phases, including lithium sulfide (Li_2_S) and nano-sized metallic particles. Accompanying this chemical transformation is a substantial volume change; for example, sulfur cathodes demonstrate a remarkable volume expansion of 78.89% upon conversion to Li_2_S, which contrasts sharply with the relatively minor 1.09% volume change observed in traditional intercalation-type cathodes [30].

**Figure 13:**
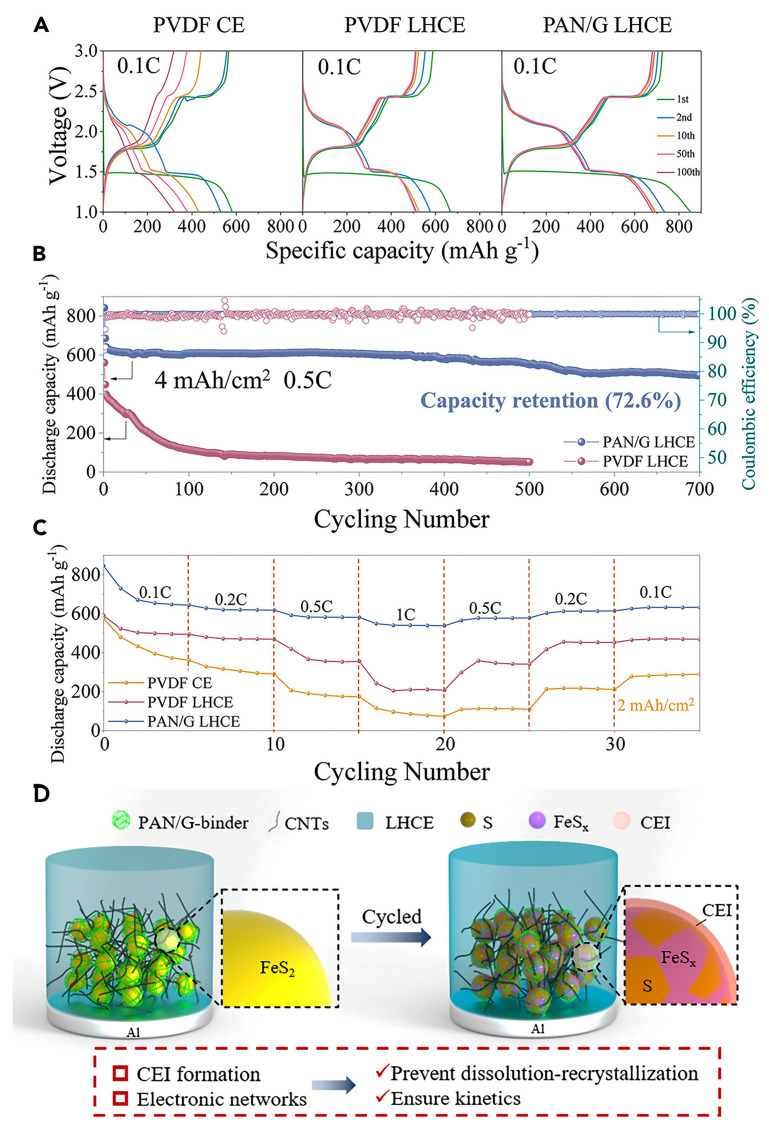
(**A**) Charge–discharge curve, (**B**) long cycling performance (at 0.5 C with an areal capacity of 4 mA hcm^−2^, (**C**) rate performances of FeS_2_ cathodes. (**D**) Illustration of the solid-electrolyte interphase formed by employing layered hydrocarbon electrolytes and three-dimensional electronic networks integrated with carbon nanotubes and polyacrylonitrile/graphene binder on the surface of FeS_2_; reproduced with permission from ref. [162], Elsevier.

To improve the durability and efficiency of these electrodes, the development of a uniform ionic-conductive solid-electrolyte interphase is critical. One effective strategy to achieve this is the use of functional electrolytes. This method helps to mitigate the dissolution–recrystallization phenomena by forming an interphase that selectively allows lithium ions to pass through while blocking other species. Notably, the application of artificial SEIs derived from vinyl carbonate on the surface of sulfur cathodes has led to substantial improvements in their cycling performance [159]. Furthermore, pyrite FeS_2_ is emerging as a highly promising option for conversion cathodes for its impressive specific capacity of 894 mA hg^−1^ and a significant energy density. Its appeal is further enhanced by its ultra-low cost, along with its favorable electronic conductivity of 10^−2^ S/cm [160]. Collectively, these attributes position FeS_2_ as a competitive candidate for advancing the development of efficient and cost-effective lithium-ion battery technologies. For example, Li et al. reveal the potential of micro-sized FeS_2_ as a conversion-type cathode for lithium–metal batteries, demonstrating remarkable longevity with three-dimensional ionic-electronic networks. By integrating a novel polymer electrolyte [161], a functional binder, and carbon nanotubes into a unified system, the micro-sized FeS_2_ cathode retains 72.6% of its capacity after 700 cycles at a current rate of 0.5 C and a density of 4 mA h/cm^2^ [162]. Creating a uniform ionic–conductive solid-electrolyte interphase (CEI) and an electronic map on FeS_2_ particles significantly enhances the electrochemistry of the conversion-type cathode by facilitating efficient lithium-ion transport and improving overall stability during cycling. A well-formed CEI not only reduces the likelihood of solvent decomposition but also helps prevent capacity loss associated with the dissolution and re-precipitation of active materials. Additionally, establishing a robust electronic network ensures excellent electron conduction within the cathode, enabling the better use of active materials during charge and discharge cycles. These enhancements collectively contribute to improved cycling stability and longevity, making the FeS_2_ cathode a more viable option for high-performance lithium-ion batteries. For instance, the development of a cathode–electrolyte interphase (CEI) as an ionic conductor is achieved using high-concentration electrolyte (LHCE). A durable electronic network is constructed by applying a functional binder and carbon nanotubes (CNTs) to the surface of the active material. This approach results in a micro-sized FeS_2_ cathode that demonstrates a notable capacity retention of 72.6% after 700 cycles at a current rate of 0.5 C, highlighting the effectiveness of these modifications in improving long-term cycling stability (as illustrated in Figure 13).

## 6. Conclusions and Future Direction

In conclusion, conversion-type cathodes present significant potential to improve the performance of lithium-ion and lithium–sulfur batteries; however, several challenges must be addressed to unlock their full capabilities in practical applications. Key issues, such as large voltage hysteresis and electrolyte decomposition reactions, result in reduced electrochemical performance due to gas generation and resistance growth. Additionally, the structural integrity of electrodes is compromised by the substantial volume changes that occur during cycling. Optimizing nanostructures is essential for minimizing lithium-ion diffusion paths and enhancing mass charge transport, which are critical factors for achieving high electrochemical performance. Moreover, the development of innovative and stable electrolyte formulations is vital to mitigate issues such as electrolyte decomposition and unfavorable interactions with cathode materials, ultimately leading to improved cycling stability and coulombic efficiency. Despite notable advancements in activating lithium sulfide (Li_2_S)-based cathodes through nanostructuring and integrating conductive carbon-based matrices or metal oxides, substantial improvements in performance, safety features, and cost-effectiveness are necessary to ensure that these technologies can compete effectively with existing lithium-ion systems.

Looking toward the future, research should focus on several key areas to facilitate the successful commercialization of conversion-type cathodes. First, advancements in synthesis techniques are crucial for producing tailored nanostructures that enhance electrochemical properties while ensuring consistency and reproducibility for large-scale manufacturing. Exploring hybridization strategies, which involve combining conversion cathodes with conductive materials, can significantly improve electron transport, mitigate resistance, and maintain structural integrity during battery cycling. Furthermore, a thorough investigation of the interactions between these cathodes and various anode materials is essential for optimizing complete cell configurations, thereby enhancing overall battery performance. Incorporating advanced characterization techniques can provide deeper insights into the mechanisms governing battery performance, allowing for targeted improvements. By addressing these strategic areas, the energy storage sector can pave the way for conversion-type cathode technologies that not only meet or exceed current performance benchmarks but also contribute to the development of innovative, safe, and efficient energy storage solutions capable of addressing the growing demands of future applications in renewable energy and electric vehicles.

## Figures and Tables

**Table 1:** Summary of nanoparticles and their respective nanocomposites used to enhance the performance of Li-S batteries.

Metal/Metal Oxide/Sulfides	Morphology	Final Form of Cathode for LiS	Initial Discharge Capacity [mA h g^−1^]	References
Fe	Mesoporous hollow carbon spheres	Fe-N/MHCS	1236_0.1 C	[140]
Pt-Ni	Nanoparticles	PtNi_C	1093.8_0.2 C	[141]
B-Co	Nanoparticles	Hollow-Co-BN-GC	1089_0.5 C	[142]
Fe-Co	Single atom on carbon nanosheets	Fe-N_C/S on Co- N_C/S (dual layer electrode)	1343_0.2 C	[143]
VS_2_	Nanoflowers	VS_2_/S	1026.6_0.2 C	[144]
	Nanoparticles	VS_2__S	1275_0.2 C	[145]
CoS_2_	Nanoparticles	CoS_2_/rGO-S	993.5_0.5 C	[146]
Co_9_S_8_	Nanosheet interconnected to form 3D	Co_9_S_8_/S75	863_2 C	[147]
NiS	Nanoparticles	S/NiS-HS	723_0.5 C	[148]
MoS_2_	Nanosheet	MoS_2_/g-C_3_N_4_/S	966_0.5 C	[149]
	Nanoparticles on carbon nanofiber (CNF)	S_MoS_2__CNF	1398_0.2 C	[150]
WS_2_	Nanosheet	C_WS_2_/S	563_2 C	[151]
FeCo_2_S_4_	Hollow nanotubes	FeCo_2_S_4_/CC_S	969_0.5 C	[152]
VSe_2_	Nanoflakes	VSe_2_-VG_CC/S	1025_0.5 C	[153]

## Data Availability

No new data were created or analyzed in this study.
